# Overexpression of *hsa-miR-30a-5p* and non-obstructive azoospermia: A case-control study

**DOI:** 10.18502/ijrm.v20i5.11054

**Published:** 2022-06-08

**Authors:** Mohammad Arefnia, Majid Motovali-Bashi, Seyed-Morteza Javadirad, Hamid Norioun

**Affiliations:** ^1^Department of Cell and Molecular Biology and Microbiology, Faculty of Biological Science and Technology, University of Isfahan, Isfahan, Iran.; ^2^Medical Genetics Department, Institute of Medical Biotechnology, National Institute of Genetic Engineering and Biotechnology (NIGEB), Tehran, Iran.

**Keywords:** Hsa-miR-30a-5p, Male infertility, KDM3A, Azoospermia, miRNA.

## Abstract

**Background:**

Some previous human and animal studies have supported the idea that *KDM3A* down-regulation might be the main cause of male infertility, especially in non-obstructive azoospermia (NOA). The regulatory role of micro-RNAs (miRNA) has been investigated in the development of male infertility.

**Objective:**

The expression level of *hsa-miR-30a-5p* in azoospermia was evaluated to reveal its possible association with the etiology of male infertility.

**Materials and Methods:**

In this case-control study, 30 men with azoospermia (19 of whom had NOA) were selected as the case individuals, and 11 men with obstructive azoospermia (OA) were selected as control individuals. The best miRNA with the strongest ability to target the* KDM3A* gene was detected via comprehensive bioinformatics analysis. Reverse transcriptase quantitative polymerase chain reaction was used to assess the expression level of *hsa-miR-30a-5p.* After analyzing the data, the expression level of *hsa-miR-30a-5p *wascompared between men with NOA and men with OA.

**Results:**

The findings supported the idea that *hsa-miR-30a-5p* is the miRNA with the best ability to target the *KDM3A* transcript. The expression analysis of *hsa-miR-30a-5p* indicated a significant overexpression (p = 0.04) in men with NOA compared to in men with OA.

**Conclusion:**

*Hsa-miR-30a-5p* was overexpressed in men with NOA compared to in control individuals. *Hsa-miR-30a-5p* could target the *KDM3A *transcript and may suppress its expression.

## 1. Introduction

Infertility is defined by the World Health Organization as an inability to conceive after at least 1 yr of regular unprotected intercourse (1). It is estimated that 15% of all couples in the world may experience infertility. Half of these failures are thought to have roots in male factors (2). Azoospermia is considered to be one of the main causes of male infertility, described as the lack of sperm in ejaculation. Azoospermia can result in infertility if an obstruction occurs in the seminiferous tubules; however, non-obstructive azoospermia (NOA) can develop independently of physical defects (3). Therefore, in people suffering from obstructive azoospermia (OA), the process of spermatogenesis is normal, but individuals with NOA show abnormal testicular spermatogenesis (4).

Animal knock-out models have supported the key role of the *KDM3A* gene for the proper development of spermatids. The main function of *KDM3A* is to demethylate H3K9s in the promoter region of the *TNP1, PRM1,* and *PRM2* genes to facilitate the expression of these downstream genes (5). Taken together, the importance of the *KDM3A* gene to maintain the proper maturation of the male germ cells seems to be undeniable (6).

Micro-RNAs (miRNAs) are single-stranded evolutionarily conserved RNA molecules with 18-25 nucleotides in length. While these small RNAs are not translated in cells, they control the function of the cells via the degradation of the other target genes during their translation (7). Few investigations have demonstrated the roles of miRNAs in the development of male infertility (8, 9). The pioneer study (conducted in 2009) introduced mir-122a as a regulatory molecule of the *Tnp2* gene (10). Other studies have highlighted the significance of miRNAs in spermatogenesis and the development of the masculine gametes (11-13).

Aberrant expression of *KDM3A* was reported previously, but the role of miRNAs controller was not discussed (5). To answer this question, *hsa-miR-30a-5p *expression in men with OA and NOA was investigated in this study.

## 2. Materials and Methods 

### Bioinformatics investigation

To find the best candidate miRNA against *KDM3A *transcription, some online databases were explored:

• miRBase (http://www.mirbase.org),

• TargetScan (http://www.targetscan.org),

• DianamicroT (http://diana.imis.athenainnovation.gr),

• miRanda (http://www.microrna.org),

• mirwalk (http://zmf.umm.uniheidelberg.de),

• MirDB (http://www.mirdb.org),

• PicTar (https://pictar.mdcberlin.de),

• MiRGen (http://carolina.imis.athenainnovation.gr)

### Sample size

Based on the prevalence of azoospermia in the population (1%) and previous studies (14, 15) the size of the sample needed was determined to be 30. The sample size was calculated by the formula: 


$$\frac{{\left(Z_{1-a\setminus 2}\right)}^{2}p\left(1-p\right)}{d^{2}}$$

The current research was performed in 2020 on 30 azoospermic tissues. Samples were collected using testicular sperm extraction and microsurgical testicular sperm extraction from men with azoospermia who were referred to the Isfahan Fertility and Infertility Center (IFIC) in Isfahan, Iran from March 2013 to March 2015. Histology clarification suggested 11 samples as OA and 19 as NOA. All men who underwent the surgery had not had any previous medical history of testicular tumor or testis-related diseases. In accordance with the process used in previous studies on this topic, OA individuals were our control group (15-17).

In accordance with the manufacturer's instructions, 50 mg of testicular tissue was immersed in RNA later stabilization reagent (Ambion Life Science, Austin, TE, USA, AM7024). The testicular tissues were preserved at 4 C for 24 hr before being homogenized with a Heidolph Homogenizer DIAX 900 (Heidolph Instruments GmbH & CO. KG, Germany).

Sinaclon (Sinaclon, Iran) RNX Plus extraction buffer was used to extract total RNA. 1% agarose gel electrophoresis and Nanodrop OneC (Thermo Scientific, USA) were used to determine the quality and quantity of extracted RNA. RT-BON (Sinaclon, Iran) adaptor primer was used to add a poly (A) tail to the end of the extracted miRNAs, and oligo (dT) primers were used for the first-strand complementary DNA synthesis. Highly specific forward and reverse primers of *hsa-miR-30a-5p* were bought from Bonyakhteh Co, Iran (http://strc.ac.ir/en). The reverse transcriptase quantitative polymerase chain reaction (RT-qPCR) was carried out in triplicate on a Choromo 4 Bio-Rad machine (USA). U6-snRNA was used as the calibrator gene (15).

### Ethical considerations

Written informed consent was obtained from all individuals. The study protocol was approved by the institutional review board and Ethics Committee of Royan Institute, Isfahan, Iran (Code: IR.ACECR.ROYAN.REC.1394.105).

### Statistical analysis

To calculate and compare the mean Cq of *hsa-miR-30a-5p* between men with OA vs. NO, *t* test were calculated using GraphPad Prism 7.05 (USA) software. P-values < 0.05 were considered significant.

## 3. Results 

### Bioinformatics exploration

Having explored various databases, we screened the existing information and *hsa-miR-30a-5p* was chosen. It was observed that *hsa-miR-30a-5p* showed the highest frequency among different databases with the highest score for the potential to impact *KDM3A *transcription.

### RNA assessment

**Figure 1:**
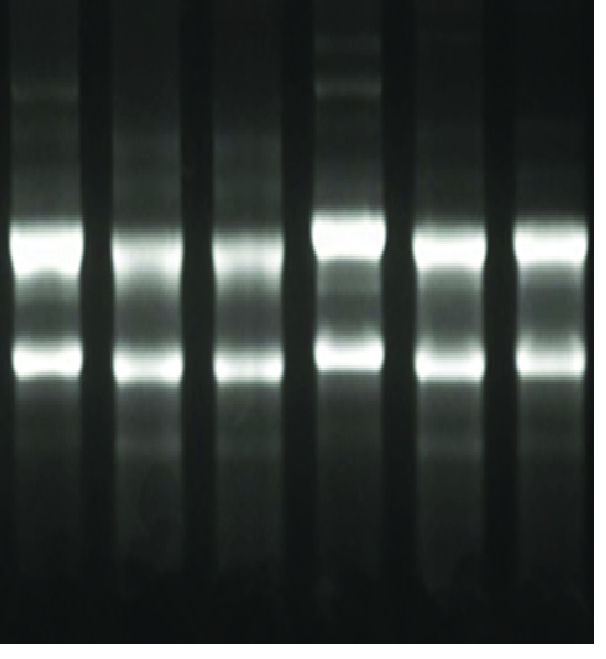
Agarose gel electrophoresis

Agarose gel electrophoresis showed a 28S rRNA band intensity 2 times greater than 18S rRNA, an indication of the appropriate handling and intactness of the extracted RNAs (Figure 1). RNA absorbance ratios of 260/280 and 260/230 were between 1/8-2, and the purity of the extracted RNAs was confirmed.

### RT-qPCR analysis 

**Figure 2:**
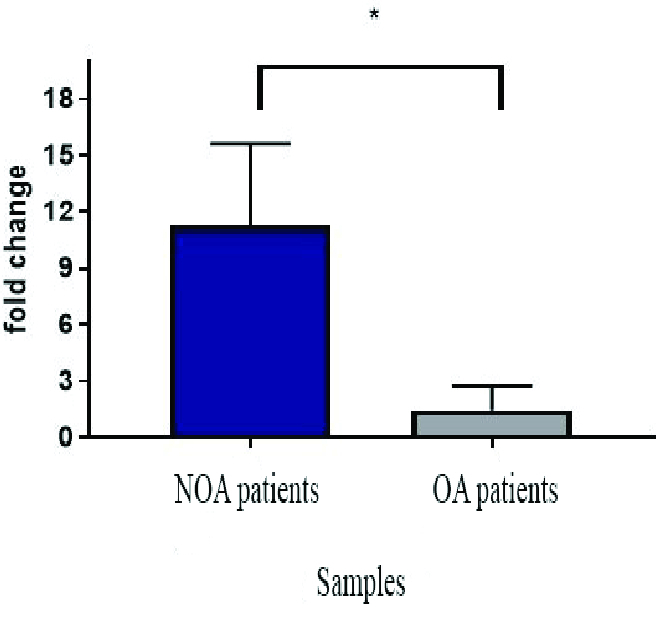
*Hsa-miR-30a-5p* expression level in men with NOA vs. in men with OA. *P < 0.05.

Melt curve analysis showed the highest specificity of the designed primer. The single pick of melt curve indicated the specificity of the primers and loss of primer dimer formation. The expression levels of *hsa-miR-30a-5p* in individuals with NOA and OA are illustrated in figure 2. Significant overexpression of *hsa-miR-30a-5p* was observed when men with NOA were compared to OA-control individuals (p = 0.04).

## 4. Discussion

In the current study, 30 testicular tissues of idiopathic azoospermia from 19 individuals with NOA and 11 individuals with OA (control) were explored. RT-qPCR data analyses indicated that the expression of *hsa-miR-30a-5p* was 11.3 times higher in men with NOA compared to OA-control individuals (p = 0.04).

In contrast, another study showed the down-regulation of *hsa-miR-30a-5p* in men with NOA. The inconsistency might be ascribed to the control individuals as they used men with testicular carcinomas as their control group (18). In accordance with our results, *hsa-miR-30a-5p* was previously identified by microarray data analysis as a suggested biomarker in asthenozoospermia (19).

Among all the different cell populations in testicular tissue, the haploid cells and late meiotic cells are the fundamental sources of miRNA production during spermatogenesis (20). The first expression of miRNAs in testicular tissue was reported in 2004 (21). Subsequently, researchers focused on finding a function of miRNAs in the physiology of the germinal duct. In 2005, for the first time, it was found that *mir-122a* caused a negative expression regulation of the *Tnp2* gene during spermatogenesis. It has also been shown that the function of this miRNA is to neutralize the expression of the gene through degradation of the gene's transcription. Also, by using the luciferase assay technique, it was revealed that 15 picomoles of *mir-122a* can reduce the gene's expression (22). A microarray data analysis revealed 154 down-regulated and 19 up-regulated miRNAs in testicular tissues and presented the first biomarkers of NOA (23). Another microarray analysis revealed 54 up-regulated and 27 down-regulated miRNAs in asthenozoospermia patients (19). Our investigation on *hsa-miR-30a-5p* suggested that overexpression of the mentioned miRNA leads to male infertility. Our study obtained similar results as another conducted on *hsa-mir-27a-3p* and its target *KDM3A*. The mentioned study revealed *hsa-mir-27a-3p* was overexpressed in NOA patients; it targeted the *KDM3A *transcript and consequently led to male infertility (15). Our study also suggested that overexpression of *hsa-miR-30a-5p* can lead to infertility in men with NOA.

Our data are contrary to a study that was conducted on prostate cancer. In that study microarray data indicated *hsa-miR-30a-5p* down-regulation occurred in NOA patients compared to prostate cancer patients. The difference in the results obtained may be due to differences in sample numbers and in the populations of the 2 studies. In that study, 3 individuals with NOA were sampled in China, but in the present study, 19 Iranian NOA patients were used for the study. Moreover, tissue heterogeneity may have led to a difference in the results of these 2 studies. The important point to be considered is the difference between the control groups of the studies. The mentioned study compared *hsa-miR-30a-5p* expression in individuals with NOA vs. in prostate cancer patients, but in the present study, the expression of miRNA in NOA patients was compared with that of OA patients (23). Hence, the results of both studies could be considered accurate.

The results of this study and a previous research show that *hsa-miR-30a-5p* can be considered as a biomarker for NOA and asthenozoospermia (19). Due to the network's expression and interconnected miRNAs, the findings can indicate a relationship between the expression of *hsa-miR-30a-5p* and the occurrence of NOA and asthenozoospermia diseases.

## 5. Conclusion

According to the current study, *hsa-miR-30a-5p* is overexpressed in NOA patients compared to OA patients, and *hsa-miR-30a-5p* could potentially target the *KDM3A* transcript and hinder it from being translated. As a result, the downstream genes *TNP1, PRM1,* and *PRM2* remain silent. All of these factors combine to induce male infertility in men with NOA.

##  Conflict of Interest

The authors declare that there is no conflict of interest.
